# Comorbidity indices in observational studies on cancer risk

**DOI:** 10.2340/1651-226X.2026.45061

**Published:** 2026-01-11

**Authors:** Björn Zethelius, Mats Talbäck, Rickard Ljung

**Affiliations:** a Use and Information Division, Swedish Medical Products Agency, Uppsala, Sweden; b Department of Public Health, Clinical Geriatrics, Uppsala University, Uppsala, Sweden; c Institute of Environmental Medicine, Karolinska Institutet, Stockholm, Sweden

**Keywords:** Multimorbidity, comorbidity, risk adjustment, indices, scoring algorithm

Dear Editor,

Comorbidity indices used in statistical models in cancer studies are essential for quantifying the burden of co-existing diseases and their impact on patient outcomes [1]. The Charlson Comorbidity Index (CCI) [2] and the Elixhauser Index (ECI) [3], which are both derived from hospitalisation data have been widely used. Recently, a Nordic Multimorbidity Index (NMI) was developed in a general population setting using health registry data from Denmark [4].

The aim of our study was to analyse comorbidity measures in relation to cancer risk. We used the Area Under the Receiver Operating Characteristic curves (AUROC) of age-and-sex alone, the NMI, the CCI and the ECI. We also explored measures based on inpatient care (number of hospitalisations, days hospitalised and number of unique diagnoses). Furthermore, we also explored the number of filled drug prescriptions (unique Anatomical Theraputic Chemical [ATC] 1 - to 7-characters codes). This was made for six outcomes: A composite of all cancers combined and five prespecified cancer diagnoses, discussed next column, using Swedish healthcare registries and cancer register [5].

Our study population comprised all persons born in 1975 or earlier who had been living in Sweden continuously from 2010 to 2014. These individuals were then monitored for cancer for a further 5 years, from 2015 to 2019, that is, before the COVID-19 pandemic. The baseline population comprised: 5,010,261 individuals with a median age of 60 years, of whom 51.4% were women. Individuals with a history of the respective malignancy within 10 years before baseline were excluded. For all cancers, 7.8% were excluded. The most common comorbidities were cardiovascular diseases (13.8%) and diabetes (5.6%). The most frequently filled prescriptions (36.8%) were cardiovascular disease preventive medicines (ATC: C07, C08 and C09).

We present AUROC with 95% confidence intervals in the two figures below and in the appendix. AUROC were calculated using 1-, 3- and 5-years look-back periods of historic data for diagnoses and a 1-year-look-back period for filled prescriptions. This was done in three models per outcome:

(a) age-and-sex alone
(b) each index and explored measures alone
(c) each index and explored measure taking age-and-sex into account

The six evaluated outcomes were the 5-years-incidences of all malignancies (ICD-10: C00-C97); Colorectal cancer (C18-C21; Lung cancer (C34), Malignant melanoma (C43); Breast cancer (C50) and Prostate cancer (C61), respectively. In the appendix we present AUROC for each outcome, in the population aged 40–64, 65–79 and 80 years and over (80-plus) years at baseline. The appendix includes 18 tables, one per outcome per age group.

Indices alone performed poorly for all malignancies in those aged 40–64 years at baseline. The NMI with a look-back period of 5 years showed the highest AUROC for lung cancer (0.604) as shown in Figure 1 and Supplementary Table 3 and ATC with 4- to 7-characters showed AUROC of 0.625 as presented in Figure 2. The lowest AUROC were observed for malignant melanoma, around 0.50 in all analyses of indices alone (see Supplementary Table 4). The highest AUROC were observed in all analyses for age-and-sex alone, ranging from 0.564 for malignant melanoma to 0.766 for lung cancer and 0.774 for prostate cancer (see Supplementary Tables 1 to 6).

**Figure 1:**
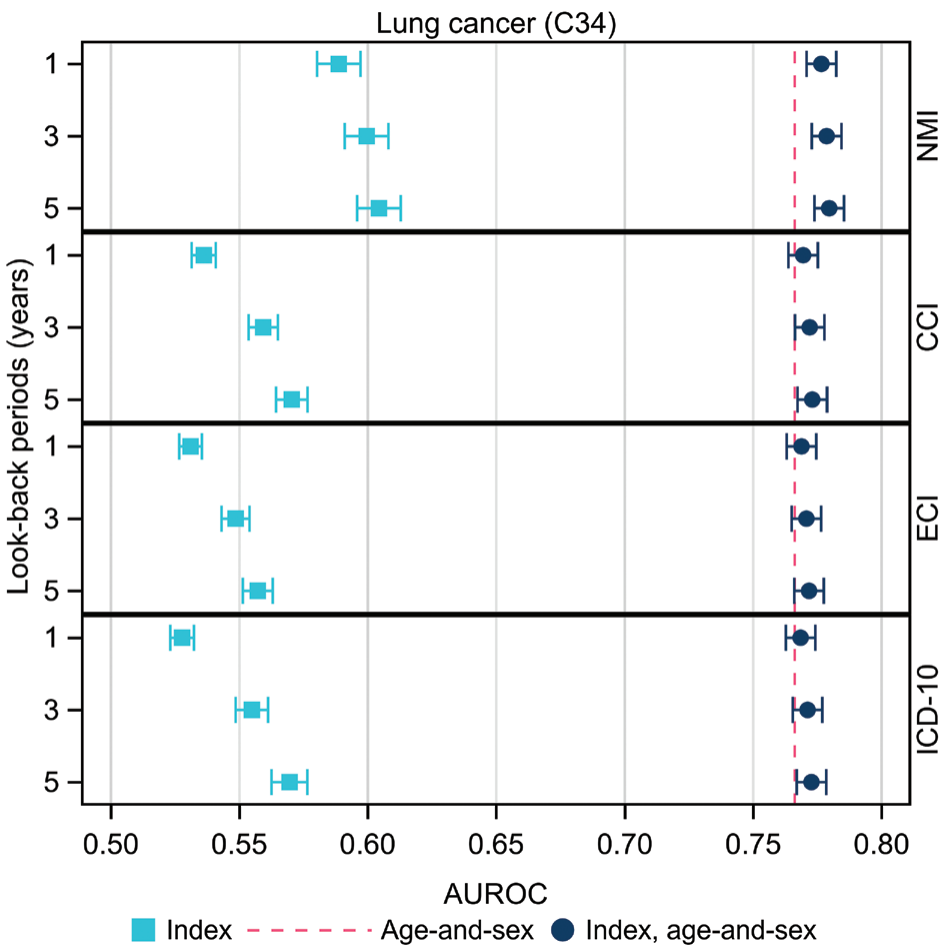
Area under the Receiver Operating Curve (AUROC) characteristics with 95% confidence intervals for 5-year follow-up in the general population, age 40–64 years free from cancer at baseline for lung cancer (ICD-10: C34) with a 1-, 3- and 5-year look-back period for diagnoses, from top to bottom, for the Nordic Multimorbidity Index (NMI), the Charlson Comorbidity Index (CCI), the Elixhauser Comorbidity Index (ECI) and numbers of distinct 3-character ICD-10 codes of main diagnoses. Models were based on (a) age-and-sex alone (The red line in each panel represents AUROC for age-and-sex), (b) index or measure alone (light blue symbols), and (c) age-and-sex and index, that is, full model (dark blue symbols).

**Figure 2:**
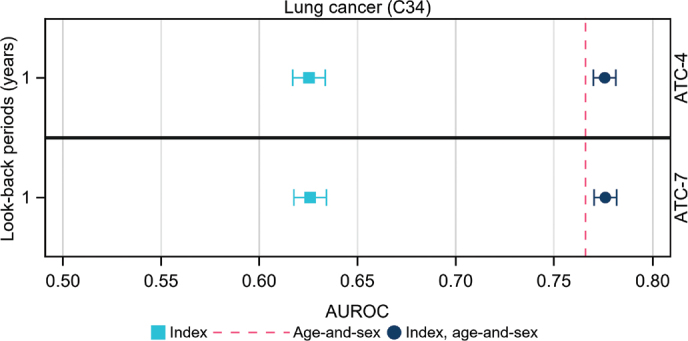
Area under the Receiver Operating Curve (AUROC) characteristics with 95% confidence intervals for a 1-year look-back period for numbers of distinct 4- and 7-character ATC-codes on filled prescriptions. Models were based on (a) age-and-sex alone (The red line in each panel represents AUROC for age-and-sex), (b) index or measure alone (light blue symbols), and (c) age-and-sex and index, that is, full model (dark blue symbols).

For lung cancer, an increasing trend in AUROC was observed with an increasing look-back period for indices and explored measures of inpatient care alone, see Figure 1 and Supplementary Table 3. For lung cancer, the AUROC was 0.766 for age-and-sex alone and, when taking age-and-sex into account, up to 0.780 for NMI and all indices or measures.

In the age group of 65–79 years, the AUROC for all indices alone (see Supplementary, Tables 7 to 12) were around 0.50 for all outcomes except for lung cancer (NMI, 0.579; ATC 4-characters, 0.563). Likewise, in the age group of 80-plus years (see Supplementary, Tables 13 to 18) AUROC for all outcomes were slightly above 0.50, except for prostate cancer (0.623 for NMI alone). Furthermore, in the age group of 80-plus years, the NMI added to age-and-sex showed an AUROC of 0.653 for prostate cancer and of 0.669 for lung cancer.

For discussion, the younger age group, the highest AUROC were observed for NMI and for filled prescriptions for lung cancer with a trend towards higher AUROC with a longer look-back period for diagnoses. Although a 1-, 3- or even a 5-year look-back period may not accurately reflect the causal period of interest for the development of many forms of cancer, filled prescriptions and/or comorbidities during these assessment periods may represent conditions of longer duration or an underlying shared risk factor that is not captured in the register data. Thus,the observed association may be considered as a marker for the exogenous risk factor of tobacco smoking for lung cancer and smoking-related comorbidities, that is, cardiovascular diseases that are captured by diagnoses and treatments incorporated into the NMI. The numbers of filled prescriptions showed AUROC of similar magnitude to that of the NMI, that is, here, this measure captures the frequently prescribed cardiovascular disease preventive medicines. For malignant melanoma, a cancer for which sunlight exposure is a unique risk factor, the AUROC was 0.50 for all indices and explored measures alone revealing no impact of comorbidities or their treatments on malign melanoma development.

Interestingly, the number of filled prescriptions alone, using a 1-year look-back period, rendered AUROC for lung cancer of a similar magnitude to that of NMI alone when, calculated using 5-years of look-back data. This suggests that the number of filled prescriptions could possibly be used as a proxy for disease burden instead of calculating complex indices. In situations where clinical background data are limited, using pharmacy dispensing data to assess comorbidity could be helpful. Furthermore, the 4-character ATC codes appear granular enough (Figure 2, Supplementary Table 3).

Here we excluded cancer at baseline and ten years before to explore comorbidity measures of cancer incidence from a population-based public health perspective. However, from the perspective of cancer treatment, comorbidities may influence outcomes such as survival, treatment tolerance, and risk of complications. Taking comorbidity into account is valuable when tailoring treatment plans, guiding shared decision-making in cancer care and stratifying patients in clinical trials. This is illustrated by a recently published study in this journal on multimorbid lung cancer patients [6]. The study showed the importance of tailoring treatment to individual patient characteristics including comorbidities using the CCI. Furthermore, in prostate cancer patients treated with external beam radiotherapy, the CCI was used, and the presence of comorbidities was observed to increase the excess risk of non-pelvic second primary cancers [7]. Thus, in different clinical and study contexts, further exploration of the NMI and other measures explored such as the number of filled prescriptions is needed to enable comparison with the most frequently used CCI. Given the range of available indices [1, 2, 3, 8], identifying the most appropriate measure to improve comorbidity adjustments is important.

The NMI was developed in a Danish population aged 40 years and over. We investigated three age groups: 40–64, 65–79 and 80-plus years to assess possible differences, given that comorbidities impact differently at young and older ages. This study was well powered statistically as can be seen on the very narrow confidence intervals presented in the figures and the tables in the Supplementary Material. Analysis of the age-group 40–64 (acting age of work-life) revealed the highest AUROC for lung cancer underlining the importance of reducing tobacco smoking to prevent cardiovascular diseases and lung cancer.

In conclusion, the AUROC for age-and-sex alone was higher than that for any other measure for all outcomes. The NMI and the ATC-codes showed slightly higher AUROC alone for lung cancer compared to the other indices or diagnose-related measures. The trend of increased AUROC with an increasing look-back period for NMI-calculations shows better capture of comorbidities related to the lung cancer risk factor of tobacco smoking. Results for filled prescriptions in the last year were like those for 5-year look back diagnostic data implying a possible use of such data as a proxy measure for comorbidity. Therefore, future studies should consider the comorbidity measure or combinations of measures that best aligns with given study populations, settings, data sources and research questions.

## Supplementary Material



## Data Availability

The data, that is, national health registry data, that support the findings of this study are available from the National Board of Health and Welfare (NBHW). Restrictions apply to the availability of these data, which were used under license for this study. Registry data can be available upon request to the NBHW (https://www.socialstyrelsen.se/en/) and Statistics Sweden (https://www.scb.se/en/).
