# Supplementary material for: Comorbidity indices in observational studies on cancer risk
Source: Acta Oncol. 2026 Jan 11;65:45061. doi: 10.2340/1651-226X.2026.45061 (PMC12800786; doi:10.2340/1651-226X.2026.45061)
Supplement: Supplementary file 1 [file AO-65-45061-s1.pdf]

Supplementary material has been published as submitted. It has not been copyedited, or typeset by Acta Oncologica

APPENDIX

Acta Oncologica

Comorbidity indices in observational studies on cancer risk

Zethelius B et al

The appendix includes 18 tables, six per age group: 40-64; 65-79; 80-plus years of age. Tables are presented in the order below for respective outcomes (ICD-10 codes) under study.

Age group 40-64 years of age

1. All carcinoma, C00-C97
2. Colorectal cancer, C18-C21
3. Lung cancer, C34
4. Malignant melanoma, C43
5. Breast cancer, C50
6. Prostate cancer, C61

Age group 65-79 years of age

7. All carcinoma, C00-C97
8. Colorectal cancer, C18-C21
9. Lung cancer, C34
10. Malignant melanoma, C43
11. Breast cancer, C50
12. Prostate cancer, C61

Age group 80-plus years of age

13. All carcinoma, C00-C97
14. Colorectal cancer, C18-C21
15. Lung cancer, C34
16. Malignant melanoma, C43
17. Breast cancer, C50
18. Prostate cancer, C61

Figure 3. Results, outcomes corresponding to tables 1 to 6 for age group 40-64 years of age.

Tables 1 to 6 are for the age group 40-64 years of age.

Table 1.

Carcinoma, all (ICD-10: C00-C97)

Index, Model, Year of Event 2015-2019		Look-back period								
		2014			2012-2014			2010-2014		
		AUROC	95% LCL	95% UCL	AUROC	95% LCL	95% UCL	AUROC	95% LCL	95% UCL
Nordic Multimorbidity Index	Index Sex Age	**0.6721**	0.6703	0.6738	**0.6722**	0.6705	0.6740	**0.6724**	0.6706	0.6742
	Sex Age	**0.6711**	0.6693	0.6728	**0.6711**	0.6693	0.6728	**0.6711**	0.6693	0.6728
	Index	**0.5142**	0.5123	0.5161	**0.5176**	0.5157	0.5195	**0.5196**	0.5177	0.5215
Charlson Comorbidity Index	Index Sex Age	**0.6717**	0.6699	0.6735	**0.6720**	0.6702	0.6738	**0.6721**	0.6703	0.6739
	Sex Age	**0.6711**	0.6693	0.6728	**0.6711**	0.6693	0.6728	**0.6711**	0.6693	0.6728
	Index	**0.5106**	0.5097	0.5114	**0.5163**	0.5152	0.5173	**0.5191**	0.5179	0.5202
Elixhauser Comorbidity Index	Index Sex Age	**0.6718**	0.6700	0.6736	**0.6720**	0.6702	0.6738	**0.6721**	0.6703	0.6739
	Sex Age	**0.6711**	0.6693	0.6728	**0.6711**	0.6693	0.6728	**0.6711**	0.6693	0.6728
	Index	**0.5098**	0.5090	0.5106	**0.5144**	0.5134	0.5154	**0.5166**	0.5155	0.5176
3-char. ICD-10 main diag.	Index Sex Age	**0.6714**	0.6696	0.6732	**0.6717**	0.6699	0.6735	**0.6719**	0.6701	0.6737
	Sex Age	**0.6711**	0.6693	0.6728	**0.6711**	0.6693	0.6728	**0.6711**	0.6693	0.6728
	Index	**0.5088**	0.5079	0.5097	**0.5183**	0.5169	0.5196	**0.5242**	0.5227	0.5257
Visits to inpatient care	Index Sex Age	**0.6713**	0.6695	0.6731	**0.6716**	0.6698	0.6734	**0.6717**	0.6699	0.6735
	Sex Age	**0.6711**	0.6693	0.6728	**0.6711**	0.6693	0.6728	**0.6711**	0.6693	0.6728
	Index	**0.5082**	0.5073	0.5091	**0.5160**	0.5147	0.5174	**0.5201**	0.5186	0.5216
Days in inpatient care	Index Sex Age	**0.6712**	0.6694	0.6730	**0.6713**	0.6695	0.6731	**0.6714**	0.6696	0.6732
	Sex Age	**0.6711**	0.6693	0.6728	**0.6711**	0.6693	0.6728	**0.6711**	0.6693	0.6728
	Index	**0.5082**	0.5073	0.5091	**0.5162**	0.5148	0.5175	**0.5203**	0.5188	0.5219
1-char. ATC	Index Sex Age	**0.6726**	0.6708	0.6744	-	-	-	-	-	-
	Sex Age	**0.6711**	0.6693	0.6728	-	-	-	-	-	-
	Index	**0.5561**	0.5542	0.5581	-	-	-	-	-	-
3-char. ATC	Index Sex Age	**0.6725**	0.6707	0.6743	-	-	-	-	-	-
	Sex Age	**0.6711**	0.6693	0.6728	-	-	-	-	-	-
	Index	**0.5587**	0.5567	0.5606	-	-	-	-	-	-
4-char. ATC	Index Sex Age	**0.6725**	0.6707	0.6743	-	-	-	-	-	-
	Sex Age	**0.6711**	0.6693	0.6728	-	-	-	-	-	-
	Index	**0.5579**	0.5559	0.5598	-	-	-	-	-	-
5-char. ATC	Index Sex Age	**0.6725**	0.6707	0.6743	-	-	-	-	-	-
	Sex Age	**0.6711**	0.6693	0.6728	-	-	-	-	-	-
	Index	**0.5576**	0.5557	0.5596	-	-	-	-	-	-
7-char. ATC	Index Sex Age	**0.6724**	0.6706	0.6742	-	-	-	-	-	-
	Sex Age	**0.6711**	0.6693	0.6728	-	-	-	-	-	-
	Index	**0.5575**	0.5555	0.5594	-	-	-	-	-	-

Table 2.

Colorectal cancer (ICD-10: C18-C21)

Index, Model, Year of Event 2015-2019		Look-back period								
		2014			2012-2014			2010-2014		
		AUROC	95% LCL	95% UCL	AUROC	95% LCL	95% UCL	AUROC	95% LCL	95% UCL
Nordic Multimorbidity Index	Index Sex Age	**0.6850**	0.6795	0.6905	**0.6850**	0.6795	0.6905	**0.6850**	0.6795	0.6905
	Sex Age	**0.6850**	0.6795	0.6905	**0.6850**	0.6795	0.6905	**0.6850**	0.6795	0.6905
	Index	**0.5028**	0.4970	0.5087	**0.5059**	0.5000	0.5117	**0.5082**	0.5023	0.5141
Charlson Comorbidity Index	Index Sex Age	**0.6851**	0.6797	0.6906	**0.6853**	0.6798	0.6907	**0.6853**	0.6799	0.6908
	Sex Age	**0.6850**	0.6795	0.6905	**0.6850**	0.6795	0.6905	**0.6850**	0.6795	0.6905
	Index	**0.5108**	0.5080	0.5136	**0.5167**	0.5133	0.5201	**0.5193**	0.5156	0.5230
Elixhauser Comorbidity Index	Index Sex Age	**0.6850**	0.6796	0.6905	**0.6851**	0.6796	0.6906	**0.6851**	0.6796	0.6906
	Sex Age	**0.6850**	0.6795	0.6905	**0.6850**	0.6795	0.6905	**0.6850**	0.6795	0.6905
	Index	**0.5103**	0.5076	0.5130	**0.5150**	0.5117	0.5183	**0.5170**	0.5134	0.5206
3-char. ICD-10 main diag.	Index Sex Age	**0.6851**	0.6796	0.6906	**0.6852**	0.6797	0.6907	**0.6852**	0.6798	0.6907
	Sex Age	**0.6850**	0.6795	0.6905	**0.6850**	0.6795	0.6905	**0.6850**	0.6795	0.6905
	Index	**0.5065**	0.5037	0.5093	**0.5153**	0.5112	0.5195	**0.5206**	0.5159	0.5253
Visits to inpatient care	Index Sex Age	**0.6851**	0.6796	0.6905	**0.6852**	0.6797	0.6907	**0.6852**	0.6797	0.6907
	Sex Age	**0.6850**	0.6795	0.6905	**0.6850**	0.6795	0.6905	**0.6850**	0.6795	0.6905
	Index	**0.5053**	0.5025	0.5082	**0.5126**	0.5085	0.5168	**0.5150**	0.5102	0.5197
Days in inpatient care	Index Sex Age	**0.6850**	0.6795	0.6905	**0.6851**	0.6797	0.6906	**0.6852**	0.6797	0.6906
	Sex Age	**0.6850**	0.6795	0.6905	**0.6850**	0.6795	0.6905	**0.6850**	0.6795	0.6905
	Index	**0.5054**	0.5025	0.5082	**0.5125**	0.5084	0.5167	**0.5148**	0.5101	0.5196
1-char. ATC	Index Sex Age	**0.6850**	0.6796	0.6905	-	-	-	-	-	-
	Sex Age	**0.6850**	0.6795	0.6905	-	-	-	-	-	-
	Index	**0.5363**	0.5303	0.5423	-	-	-	-	-	-
3-char. ATC	Index Sex Age	**0.6851**	0.6796	0.6905	-	-	-	-	-	-
	Sex Age	**0.6850**	0.6795	0.6905	-	-	-	-	-	-
	Index	**0.5407**	0.5346	0.5468	-	-	-	-	-	-
4-char. ATC	Index Sex Age	**0.6851**	0.6796	0.6906	-	-	-	-	-	-
	Sex Age	**0.6850**	0.6795	0.6905	-	-	-	-	-	-
	Index	**0.5400**	0.5339	0.5461	-	-	-	-	-	-
5-char. ATC	Index Sex Age	**0.6851**	0.6796	0.6906	-	-	-	-	-	-
	Sex Age	**0.6850**	0.6795	0.6905	-	-	-	-	-	-
	Index	**0.5400**	0.5339	0.5461	-	-	-	-	-	-
7-char. ATC	Index Sex Age	**0.6851**	0.6796	0.6906	-	-	-	-	-	-
	Sex Age	**0.6850**	0.6795	0.6905	-	-	-	-	-	-
	Index	**0.5396**	0.5335	0.5457	-	-	-	-	-	-

Table 3.

Lung cancer (ICD-10: C34)

Index, Model, Year of Event 2015-2019		Look-back period								
		2014			2012-2014			2010-2014		
		AUROC	95% LCL	95% UCL	AUROC	95% LCL	95% UCL	AUROC	95% LCL	95% UCL
Nordic Multimorbidity Index	Index Sex Age	**0.7766**	0.7708	0.7823	**0.7786**	0.7728	0.7844	**0.7796**	0.7739	0.7854
	Sex Age	**0.7662**	0.7604	0.7719	**0.7662**	0.7604	0.7719	**0.7662**	0.7604	0.7719
	Index	**0.5887**	0.5802	0.5972	**0.5995**	0.5910	0.6080	**0.6043**	0.5958	0.6128
Charlson Comorbidity Index	Index Sex Age	**0.7695**	0.7637	0.7752	**0.7720**	0.7663	0.7778	**0.7730**	0.7673	0.7788
	Sex Age	**0.7662**	0.7604	0.7719	**0.7662**	0.7604	0.7719	**0.7662**	0.7604	0.7719
	Index	**0.5362**	0.5315	0.5409	**0.5593**	0.5536	0.5650	**0.5704**	0.5643	0.5766
Elixhauser Comorbidity Index	Index Sex Age	**0.7688**	0.7631	0.7746	**0.7707**	0.7650	0.7765	**0.7718**	0.7660	0.7775
	Sex Age	**0.7662**	0.7604	0.7719	**0.7662**	0.7604	0.7719	**0.7662**	0.7604	0.7719
	Index	**0.5310**	0.5266	0.5355	**0.5485**	0.5431	0.5539	**0.5572**	0.5513	0.5630
3-char. ICD-10 main diag.	Index Sex Age	**0.7685**	0.7627	0.7742	**0.7712**	0.7655	0.7769	**0.7727**	0.7670	0.7784
	Sex Age	**0.7662**	0.7604	0.7719	**0.7662**	0.7604	0.7719	**0.7662**	0.7604	0.7719
	Index	**0.5277**	0.5231	0.5323	**0.5548**	0.5486	0.5611	**0.5695**	0.5625	0.5765
Visits to inpatient care	Index Sex Age	**0.7677**	0.7619	0.7734	**0.7688**	0.7630	0.7745	**0.7694**	0.7637	0.7751
	Sex Age	**0.7662**	0.7604	0.7719	**0.7662**	0.7604	0.7719	**0.7662**	0.7604	0.7719
	Index	**0.5266**	0.5220	0.5312	**0.5515**	0.5452	0.5579	**0.5630**	0.5559	0.5701
Days in inpatient care	Index Sex Age	**0.7668**	0.7610	0.7725	**0.7675**	0.7618	0.7733	**0.7682**	0.7625	0.7740
	Sex Age	**0.7662**	0.7604	0.7719	**0.7662**	0.7604	0.7719	**0.7662**	0.7604	0.7719
	Index	**0.5267**	0.5220	0.5313	**0.5517**	0.5453	0.5581	**0.5633**	0.5562	0.5705
1-char. ATC	Index Sex Age	**0.7738**	0.7681	0.7795	-	-	-	-	-	-
	Sex Age	**0.7662**	0.7604	0.7719	-	-	-	-	-	-
	Index	**0.6174**	0.6093	0.6256	-	-	-	-	-	-
3-char. ATC	Index Sex Age	**0.7755**	0.7698	0.7812	-	-	-	-	-	-
	Sex Age	**0.7662**	0.7604	0.7719	-	-	-	-	-	-
	Index	**0.6242**	0.6160	0.6325	-	-	-	-	-	-
4-char. ATC	Index Sex Age	**0.7759**	0.7702	0.7816	-	-	-	-	-	-
	Sex Age	**0.7662**	0.7604	0.7719	-	-	-	-	-	-
	Index	**0.6253**	0.6170	0.6336	-	-	-	-	-	-
5-char. ATC	Index Sex Age	**0.7762**	0.7705	0.7819	-	-	-	-	-	-
	Sex Age	**0.7662**	0.7604	0.7719	-	-	-	-	-	-
	Index	**0.6261**	0.6179	0.6344	-	-	-	-	-	-
7-char. ATC	Index Sex Age	**0.7762**	0.7705	0.7819	-	-	-	-	-	-
	Sex Age	**0.7662**	0.7604	0.7719	-	-	-	-	-	-
	Index	**0.6259**	0.6176	0.6342	-	-	-	-	-	-

Table 4.

Malignant melanoma of skin (ICD-10: C43)

Index, Model, Year of Event 2015-2019		Look-back period								
		2014			2012-2014			2010-2014		
		AUROC	95% LCL	95% UCL	AUROC	95% LCL	95% UCL	AUROC	95% LCL	95% UCL
Nordic Multimorbidity Index	Index Sex Age	**0.5662**	0.5599	0.5725	**0.5661**	0.5597	0.5724	**0.5667**	0.5603	0.5730
	Sex Age	**0.5641**	0.5577	0.5704	**0.5641**	0.5577	0.5704	**0.5641**	0.5577	0.5704
	Index	**0.5081**	0.5023	0.5138	**0.5083**	0.5026	0.5141	**0.5085**	0.5027	0.5143
Charlson Comorbidity Index	Index Sex Age	**0.5641**	0.5578	0.5704	**0.5641**	0.5578	0.5704	**0.5639**	0.5576	0.5703
	Sex Age	**0.5641**	0.5577	0.5704	**0.5641**	0.5577	0.5704	**0.5641**	0.5577	0.5704
	Index	**0.5035**	0.5008	0.5062	**0.5044**	0.5011	0.5077	**0.5040**	0.5004	0.5076
Elixhauser Comorbidity Index	Index Sex Age	**0.5641**	0.5578	0.5705	**0.5640**	0.5577	0.5703	**0.5640**	0.5577	0.5703
	Sex Age	**0.5641**	0.5577	0.5704	**0.5641**	0.5577	0.5704	**0.5641**	0.5577	0.5704
	Index	**0.5025**	0.4999	0.5050	**0.5046**	0.5014	0.5078	**0.5054**	0.5019	0.5089
3-char. ICD-10 main diag.	Index Sex Age	**0.5644**	0.5581	0.5707	**0.5644**	0.5581	0.5707	**0.5646**	0.5583	0.5710
	Sex Age	**0.5641**	0.5577	0.5704	**0.5641**	0.5577	0.5704	**0.5641**	0.5577	0.5704
	Index	**0.5009**	0.4982	0.5036	**0.4997**	0.4956	0.5037	**0.4982**	0.4935	0.5029
Visits to inpatient care	Index Sex Age	**0.5643**	0.5580	0.5706	**0.5642**	0.5579	0.5705	**0.5645**	0.5582	0.5708
	Sex Age	**0.5641**	0.5577	0.5704	**0.5641**	0.5577	0.5704	**0.5641**	0.5577	0.5704
	Index	**0.5004**	0.4976	0.5033	**0.5005**	0.4963	0.5047	**0.4999**	0.4950	0.5047
Days in inpatient care	Index Sex Age	**0.5643**	0.5580	0.5706	**0.5643**	0.5580	0.5706	**0.5646**	0.5583	0.5709
	Sex Age	**0.5641**	0.5577	0.5704	**0.5641**	0.5577	0.5704	**0.5641**	0.5577	0.5704
	Index	**0.5004**	0.4976	0.5032	**0.5003**	0.4961	0.5045	**0.4993**	0.4945	0.5042
1-char. ATC	Index Sex Age	**0.5645**	0.5582	0.5708	-	-	-	-	-	-
	Sex Age	**0.5641**	0.5577	0.5704	-	-	-	-	-	-
	Index	**0.5103**	0.5040	0.5167	-	-	-	-	-	-
3-char. ATC	Index Sex Age	**0.5650**	0.5587	0.5713	-	-	-	-	-	-
	Sex Age	**0.5641**	0.5577	0.5704	-	-	-	-	-	-
	Index	**0.5096**	0.5032	0.5159	-	-	-	-	-	-
4-char. ATC	Index Sex Age	**0.5652**	0.5589	0.5715	-	-	-	-	-	-
	Sex Age	**0.5641**	0.5577	0.5704	-	-	-	-	-	-
	Index	**0.5088**	0.5024	0.5151	-	-	-	-	-	-
5-char. ATC	Index Sex Age	**0.5653**	0.5590	0.5716	-	-	-	-	-	-
	Sex Age	**0.5641**	0.5577	0.5704	-	-	-	-	-	-
	Index	**0.5086**	0.5023	0.5150	-	-	-	-	-	-
7-char. ATC	Index Sex Age	**0.5653**	0.5590	0.5716	-	-	-	-	-	-
	Sex Age	**0.5641**	0.5577	0.5704	-	-	-	-	-	-
	Index	**0.5087**	0.5024	0.5150	-	-	-	-	-	-

Table 5.

Breast cancer (ICD-10: C50)

Index, Model, Year of Event 2015-2019		Look-back period								
		2014			2012-2014			2010-2014		
		AUROC	95% LCL	95% UCL	AUROC	95% LCL	95% UCL	AUROC	95% LCL	95% UCL
Nordic Multimorbidity Index	Index Sex Age	**0.5920**	0.5878	0.5963	**0.5920**	0.5878	0.5963	**0.5920**	0.5877	0.5962
	Sex Age	**0.5917**	0.5874	0.5959	**0.5917**	0.5874	0.5959	**0.5917**	0.5874	0.5959
	Index	**0.5017**	0.4977	0.5057	**0.5014**	0.4973	0.5054	**0.5000**	0.5000	0.5000
Charlson Comorbidity Index	Index Sex Age	**0.5921**	0.5878	0.5964	**0.5919**	0.5877	0.5962	**0.5918**	0.5876	0.5961
	Sex Age	**0.5917**	0.5874	0.5959	**0.5917**	0.5874	0.5959	**0.5917**	0.5874	0.5959
	Index	**0.5005**	0.4988	0.5021	**0.5007**	0.4986	0.5028	**0.5017**	0.4994	0.5040
Elixhauser Comorbidity Index	Index Sex Age	**0.5918**	0.5876	0.5961	**0.5919**	0.5876	0.5961	**0.5918**	0.5876	0.5961
	Sex Age	**0.5917**	0.5874	0.5959	**0.5917**	0.5874	0.5959	**0.5917**	0.5874	0.5959
	Index	**0.5013**	0.4998	0.5029	**0.5025**	0.5005	0.5045	**0.5033**	0.5010	0.5055
3-char. ICD-10 main diag.	Index Sex Age	**0.5919**	0.5877	0.5962	**0.5920**	0.5877	0.5962	**0.5920**	0.5877	0.5962
	Sex Age	**0.5917**	0.5874	0.5959	**0.5917**	0.5874	0.5959	**0.5917**	0.5874	0.5959
	Index	**0.5008**	0.4990	0.5027	**0.4993**	0.4966	0.5020	**0.4981**	0.4949	0.5013
Visits to inpatient care	Index Sex Age	**0.5920**	0.5877	0.5962	**0.5919**	0.5877	0.5962	**0.5918**	0.5876	0.5961
	Sex Age	**0.5917**	0.5874	0.5959	**0.5917**	0.5874	0.5959	**0.5917**	0.5874	0.5959
	Index	**0.5023**	0.5004	0.5041	**0.5035**	0.5007	0.5063	**0.5045**	0.5012	0.5078
Days in inpatient care	Index Sex Age	**0.5920**	0.5877	0.5962	**0.5920**	0.5878	0.5962	**0.5919**	0.5876	0.5961
	Sex Age	**0.5917**	0.5874	0.5959	**0.5917**	0.5874	0.5959	**0.5917**	0.5874	0.5959
	Index	**0.5023**	0.5004	0.5042	**0.5034**	0.5005	0.5062	**0.5043**	0.5010	0.5077
1-char. ATC	Index Sex Age	**0.5918**	0.5875	0.5960	-	-	-	-	-	-
	Sex Age	**0.5917**	0.5874	0.5959	-	-	-	-	-	-
	Index	**0.5166**	0.5123	0.5209	-	-	-	-	-	-
3-char. ATC	Index Sex Age	**0.5916**	0.5874	0.5959	-	-	-	-	-	-
	Sex Age	**0.5917**	0.5874	0.5959	-	-	-	-	-	-
	Index	**0.5174**	0.5131	0.5217	-	-	-	-	-	-
4-char. ATC	Index Sex Age	**0.5917**	0.5874	0.5959	-	-	-	-	-	-
	Sex Age	**0.5917**	0.5874	0.5959	-	-	-	-	-	-
	Index	**0.5168**	0.5125	0.5212	-	-	-	-	-	-
5-char. ATC	Index Sex Age	**0.5917**	0.5874	0.5959	-	-	-	-	-	-
	Sex Age	**0.5917**	0.5874	0.5959	-	-	-	-	-	-
	Index	**0.5166**	0.5123	0.5209	-	-	-	-	-	-
7-char. ATC	Index Sex Age	**0.5917**	0.5874	0.5959	-	-	-	-	-	-
	Sex Age	**0.5917**	0.5874	0.5959	-	-	-	-	-	-
	Index	**0.5165**	0.5122	0.5208	-	-	-	-	-	-

Table 6.

Prostate cancer (ICD-10: C61)

Index, Model, Year of Event 2015-2019		Look-back period								
		2014			2012-2014			2010-2014		
		AUROC	95% LCL	95% UCL	AUROC	95% LCL	95% UCL	AUROC	95% LCL	95% UCL
Nordic Multimorbidity Index	Index Sex Age	**0.7757**	0.7729	0.7785	**0.7758**	0.7730	0.7786	**0.7759**	0.7731	0.7787
	Sex Age	**0.7744**	0.7716	0.7772	**0.7744**	0.7716	0.7772	**0.7744**	0.7716	0.7772
	Index	**0.5123**	0.5082	0.5164	**0.5093**	0.5051	0.5134	**0.5076**	0.5035	0.5118
Charlson Comorbidity Index	Index Sex Age	**0.7749**	0.7721	0.7777	**0.7750**	0.7722	0.7778	**0.7750**	0.7722	0.7778
	Sex Age	**0.7744**	0.7716	0.7772	**0.7744**	0.7716	0.7772	**0.7744**	0.7716	0.7772
	Index	**0.5054**	0.5036	0.5072	**0.5100**	0.5077	0.5122	**0.5130**	0.5105	0.5155
Elixhauser Comorbidity Index	Index Sex Age	**0.7750**	0.7722	0.7778	**0.7752**	0.7724	0.7780	**0.7751**	0.7723	0.7779
	Sex Age	**0.7744**	0.7716	0.7772	**0.7744**	0.7716	0.7772	**0.7744**	0.7716	0.7772
	Index	**0.5049**	0.5031	0.5066	**0.5080**	0.5059	0.5102	**0.5102**	0.5078	0.5125
3-char. ICD-10 main diag.	Index Sex Age	**0.7746**	0.7718	0.7774	**0.7746**	0.7718	0.7775	**0.7747**	0.7719	0.7775
	Sex Age	**0.7744**	0.7716	0.7772	**0.7744**	0.7716	0.7772	**0.7744**	0.7716	0.7772
	Index	**0.5064**	0.5044	0.5084	**0.5171**	0.5142	0.5200	**0.5229**	0.5196	0.5262
Visits to inpatient care	Index Sex Age	**0.7747**	0.7719	0.7775	**0.7747**	0.7719	0.7775	**0.7747**	0.7719	0.7775
	Sex Age	**0.7744**	0.7716	0.7772	**0.7744**	0.7716	0.7772	**0.7744**	0.7716	0.7772
	Index	**0.5066**	0.5046	0.5086	**0.5175**	0.5145	0.5204	**0.5234**	0.5201	0.5267
Days in inpatient care	Index Sex Age	**0.7747**	0.7719	0.7775	**0.7748**	0.7720	0.7776	**0.7748**	0.7720	0.7776
	Sex Age	**0.7744**	0.7716	0.7772	**0.7744**	0.7716	0.7772	**0.7744**	0.7716	0.7772
	Index	**0.4934**	0.4914	0.4954	**0.4825**	0.4796	0.4854	**0.4766**	0.4732	0.4799
1-char. ATC	Index Sex Age	**0.7749**	0.7721	0.7777	-	-	-	-	-	-
	Sex Age	**0.7744**	0.7716	0.7772	-	-	-	-	-	-
	Index	**0.5770**	0.5729	0.5812	-	-	-	-	-	-
3-char. ATC	Index Sex Age	**0.7740**	0.7712	0.7768	-	-	-	-	-	-
	Sex Age	**0.7744**	0.7716	0.7772	-	-	-	-	-	-
	Index	**0.5779**	0.5738	0.5821	-	-	-	-	-	-
4-char. ATC	Index Sex Age	**0.7741**	0.7712	0.7769	-	-	-	-	-	-
	Sex Age	**0.7744**	0.7716	0.7772	-	-	-	-	-	-
	Index	**0.5761**	0.5720	0.5803	-	-	-	-	-	-
5-char. ATC	Index Sex Age	**0.7741**	0.7713	0.7769	-	-	-	-	-	-
	Sex Age	**0.7744**	0.7716	0.7772	-	-	-	-	-	-
	Index	**0.5752**	0.5711	0.5794	-	-	-	-	-	-
7-char. ATC	Index Sex Age	**0.7741**	0.7713	0.7769	-	-	-	-	-	-
	Sex Age	**0.7744**	0.7716	0.7772	-	-	-	-	-	-
	Index	**0.5752**	0.5710	0.5793	-	-	-	-	-	-

Tables 7 to 12 are for the age group 65-79 years of age.

Table 7.

Carcinoma, all (ICD-10: C00-C97)

Index, Model, Year of Event 2015-2019		Look-back period								
		2014			2012-2014			2010-2014		
		AUROC	95% LCL	95% UCL	AUROC	95% LCL	95% UCL	AUROC	95% LCL	95% UCL
Nordic Multimorbidity Index	Index Sex Age	**0.5723**	0.5706	0.5740	**0.5722**	0.5705	0.5739	**0.5722**	0.5705	0.5739
	Sex Age	**0.5722**	0.5705	0.5739	**0.5722**	0.5705	0.5739	**0.5722**	0.5705	0.5739
	Index	**0.4993**	0.4977	0.5010	**0.4961**	0.4944	0.4977	**0.5055**	0.5039	0.5072
Charlson Comorbidity Index	Index Sex Age	**0.5723**	0.5706	0.5740	**0.5723**	0.5707	0.5740	**0.5724**	0.5707	0.5740
	Sex Age	**0.5722**	0.5705	0.5739	**0.5722**	0.5705	0.5739	**0.5722**	0.5705	0.5739
	Index	**0.5047**	0.5037	0.5057	**0.5082**	0.5070	0.5093	**0.5101**	0.5088	0.5114
Elixhauser Comorbidity Index	Index Sex Age	**0.5721**	0.5704	0.5738	**0.5724**	0.5707	0.5741	**0.5724**	0.5708	0.5741
	Sex Age	**0.5722**	0.5705	0.5739	**0.5722**	0.5705	0.5739	**0.5722**	0.5705	0.5739
	Index	**0.5062**	0.5052	0.5071	**0.5100**	0.5089	0.5112	**0.5118**	0.5105	0.5130
3-char. ICD-10 main diag.	Index Sex Age	**0.5724**	0.5707	0.5740	**0.5722**	0.5705	0.5738	**0.5721**	0.5704	0.5738
	Sex Age	**0.5722**	0.5705	0.5739	**0.5722**	0.5705	0.5739	**0.5722**	0.5705	0.5739
	Index	**0.5021**	0.5011	0.5030	**0.5067**	0.5054	0.5081	**0.5089**	0.5074	0.5103
Visits to inpatient care	Index Sex Age	**0.5724**	0.5707	0.5741	**0.5722**	0.5705	0.5739	**0.5722**	0.5705	0.5738
	Sex Age	**0.5722**	0.5705	0.5739	**0.5722**	0.5705	0.5739	**0.5722**	0.5705	0.5739
	Index	**0.4979**	0.4970	0.4989	**0.5066**	0.5053	0.5080	**0.5089**	0.5074	0.5103
Days in inpatient care	Index Sex Age	**0.5726**	0.5709	0.5743	**0.5726**	0.5709	0.5743	**0.5726**	0.5709	0.5743
	Sex Age	**0.5722**	0.5705	0.5739	**0.5722**	0.5705	0.5739	**0.5722**	0.5705	0.5739
	Index	**0.4980**	0.4971	0.4990	**0.4937**	0.4923	0.4950	**0.4916**	0.4901	0.4930
1-char. ATC	Index Sex Age	**0.5742**	0.5726	0.5759	-	-	-	-	-	-
	Sex Age	**0.5722**	0.5705	0.5739	-	-	-	-	-	-
	Index	**0.5174**	0.5157	0.5190	-	-	-	-	-	-
3-char. ATC	Index Sex Age	**0.5740**	0.5723	0.5757	-	-	-	-	-	-
	Sex Age	**0.5722**	0.5705	0.5739	-	-	-	-	-	-
	Index	**0.5194**	0.5177	0.5211	-	-	-	-	-	-
4-char. ATC	Index Sex Age	**0.5739**	0.5723	0.5756	-	-	-	-	-	-
	Sex Age	**0.5722**	0.5705	0.5739	-	-	-	-	-	-
	Index	**0.5192**	0.5176	0.5209	-	-	-	-	-	-
5-char. ATC	Index Sex Age	**0.5738**	0.5721	0.5754	-	-	-	-	-	-
	Sex Age	**0.5722**	0.5705	0.5739	-	-	-	-	-	-
	Index	**0.5191**	0.5175	0.5208	-	-	-	-	-	-
7-char. ATC	Index Sex Age	**0.5737**	0.5720	0.5754	-	-	-	-	-	-
	Sex Age	**0.5722**	0.5705	0.5739	-	-	-	-	-	-
	Index	**0.5189**	0.5173	0.5206	-	-	-	-	-	-

Table 8.

Colorectal cancer (ICD-10: C18-C21)

Index, Model, Year of Event 2015-2019		Look-back period								
		2014			2012-2014			2010-2014		
		AUROC	95% LCL	95% UCL	AUROC	95% LCL	95% UCL	AUROC	95% LCL	95% UCL
Nordic Multimorbidity Index	Index Sex Age	**0.5812**	0.5769	0.5855	**0.5808**	0.5765	0.5851	**0.5805**	0.5762	0.5848
	Sex Age	**0.5792**	0.5749	0.5834	**0.5792**	0.5749	0.5834	**0.5792**	0.5749	0.5834
	Index	**0.5042**	0.4999	0.5085	**0.5011**	0.4968	0.5054	**0.4993**	0.4950	0.5036
Charlson Comorbidity Index	Index Sex Age	**0.5800**	0.5758	0.5843	**0.5796**	0.5753	0.5839	**0.5795**	0.5752	0.5838
	Sex Age	**0.5792**	0.5749	0.5834	**0.5792**	0.5749	0.5834	**0.5792**	0.5749	0.5834
	Index	**0.4991**	0.4964	0.5018	**0.5062**	0.5029	0.5095	**0.5086**	0.5051	0.5121
Elixhauser Comorbidity Index	Index Sex Age	**0.5800**	0.5758	0.5843	**0.5795**	0.5752	0.5838	**0.5795**	0.5752	0.5837
	Sex Age	**0.5792**	0.5749	0.5834	**0.5792**	0.5749	0.5834	**0.5792**	0.5749	0.5834
	Index	**0.4976**	0.4949	0.5003	**0.5079**	0.5047	0.5111	**0.5107**	0.5073	0.5141
3-char. ICD-10 main diag.	Index Sex Age	**0.5798**	0.5756	0.5841	**0.5801**	0.5759	0.5844	**0.5801**	0.5759	0.5844
	Sex Age	**0.5792**	0.5749	0.5834	**0.5792**	0.5749	0.5834	**0.5792**	0.5749	0.5834
	Index	**0.5006**	0.4981	0.5032	**0.4981**	0.4946	0.5016	**0.4976**	0.4938	0.5014
Visits to inpatient care	Index Sex Age	**0.5800**	0.5757	0.5842	**0.5801**	0.5758	0.5844	**0.5800**	0.5758	0.5843
	Sex Age	**0.5792**	0.5749	0.5834	**0.5792**	0.5749	0.5834	**0.5792**	0.5749	0.5834
	Index	**0.5007**	0.4981	0.5033	**0.4981**	0.4946	0.5016	**0.4977**	0.4938	0.5015
Days in inpatient care	Index Sex Age	**0.5802**	0.5760	0.5845	**0.5803**	0.5760	0.5845	**0.5802**	0.5759	0.5845
	Sex Age	**0.5792**	0.5749	0.5834	**0.5792**	0.5749	0.5834	**0.5792**	0.5749	0.5834
	Index	**0.5008**	0.4982	0.5034	**0.4977**	0.4942	0.5012	**0.4970**	0.4932	0.5009
1-char. ATC	Index Sex Age	**0.5802**	0.5760	0.5845	-	-	-	-	-	-
	Sex Age	**0.5792**	0.5749	0.5834	-	-	-	-	-	-
	Index	**0.5011**	0.4968	0.5053	-	-	-	-	-	-
3-char. ATC	Index Sex Age	**0.5799**	0.5756	0.5841	-	-	-	-	-	-
	Sex Age	**0.5792**	0.5749	0.5834	-	-	-	-	-	-
	Index	**0.5052**	0.5009	0.5095	-	-	-	-	-	-
4-char. ATC	Index Sex Age	**0.5798**	0.5756	0.5841	-	-	-	-	-	-
	Sex Age	**0.5792**	0.5749	0.5834	-	-	-	-	-	-
	Index	**0.5049**	0.5006	0.5092	-	-	-	-	-	-
5-char. ATC	Index Sex Age	**0.5798**	0.5756	0.5841	-	-	-	-	-	-
	Sex Age	**0.5792**	0.5749	0.5834	-	-	-	-	-	-
	Index	**0.5000**	0.5000	0.5000	-	-	-	-	-	-
7-char. ATC	Index Sex Age	**0.5799**	0.5757	0.5842	-	-	-	-	-	-
	Sex Age	**0.5792**	0.5749	0.5834	-	-	-	-	-	-
	Index	**0.4958**	0.4915	0.5000	-	-	-	-	-	-

Table 9.

Lung cancer (ICD-10: C34)

Index, Model, Year of Event 2015-2019		Look-back period								
		2014			2012-2014			2010-2014		
		AUROC	95% LCL	95% UCL	AUROC	95% LCL	95% UCL	AUROC	95% LCL	95% UCL
Nordic Multimorbidity Index	Index Sex Age	**0.5722**	0.5674	0.5769	**0.5805**	0.5758	0.5853	**0.5851**	0.5803	0.5899
	Sex Age	**0.5424**	0.5377	0.5472	**0.5424**	0.5377	0.5472	**0.5424**	0.5377	0.5472
	Index	**0.5639**	0.5589	0.5688	**0.5735**	0.5685	0.5784	**0.5790**	0.5741	0.5840
Charlson Comorbidity Index	Index Sex Age	**0.5521**	0.5474	0.5568	**0.5616**	0.5568	0.5664	**0.5669**	0.5621	0.5717
	Sex Age	**0.5424**	0.5377	0.5472	**0.5424**	0.5377	0.5472	**0.5424**	0.5377	0.5472
	Index	**0.5257**	0.5223	0.5292	**0.5426**	0.5385	0.5467	**0.5514**	0.5471	0.5557
Elixhauser Comorbidity Index	Index Sex Age	**0.5460**	0.5413	0.5507	**0.5520**	0.5472	0.5567	**0.5549**	0.5501	0.5596
	Sex Age	**0.5424**	0.5377	0.5472	**0.5424**	0.5377	0.5472	**0.5424**	0.5377	0.5472
	Index	**0.5191**	0.5158	0.5224	**0.5335**	0.5296	0.5374	**0.5393**	0.5351	0.5434
3-char. ICD-10 main diag.	Index Sex Age	**0.5463**	0.5416	0.5510	**0.5518**	0.5471	0.5565	**0.5550**	0.5502	0.5597
	Sex Age	**0.5424**	0.5377	0.5472	**0.5424**	0.5377	0.5472	**0.5424**	0.5377	0.5472
	Index	**0.5130**	0.5098	0.5162	**0.5271**	0.5229	0.5313	**0.5352**	0.5307	0.5398
Visits to inpatient care	Index Sex Age	**0.5446**	0.5399	0.5493	**0.5488**	0.5441	0.5535	**0.5506**	0.5459	0.5553
	Sex Age	**0.5424**	0.5377	0.5472	**0.5424**	0.5377	0.5472	**0.5424**	0.5377	0.5472
	Index	**0.5129**	0.5097	0.5161	**0.5268**	0.5226	0.5311	**0.5354**	0.5309	0.5400
Days in inpatient care	Index Sex Age	**0.5428**	0.5381	0.5475	**0.5445**	0.5398	0.5492	**0.5458**	0.5411	0.5505
	Sex Age	**0.5424**	0.5377	0.5472	**0.5424**	0.5377	0.5472	**0.5424**	0.5377	0.5472
	Index	**0.5129**	0.5097	0.5161	**0.5273**	0.5231	0.5316	**0.5363**	0.5317	0.5409
1-char. ATC	Index Sex Age	**0.5637**	0.5588	0.5685	-	-	-	-	-	-
	Sex Age	**0.5424**	0.5377	0.5472	-	-	-	-	-	-
	Index	**0.5554**	0.5505	0.5603	-	-	-	-	-	-
3-char. ATC	Index Sex Age	**0.5682**	0.5633	0.5730	-	-	-	-	-	-
	Sex Age	**0.5424**	0.5377	0.5472	-	-	-	-	-	-
	Index	**0.5601**	0.5551	0.5651	-	-	-	-	-	-
4-char. ATC	Index Sex Age	**0.5707**	0.5658	0.5755	-	-	-	-	-	-
	Sex Age	**0.5424**	0.5377	0.5472	-	-	-	-	-	-
	Index	**0.5630**	0.5580	0.5679	-	-	-	-	-	-
5-char. ATC	Index Sex Age	**0.5705**	0.5656	0.5753	-	-	-	-	-	-
	Sex Age	**0.5424**	0.5377	0.5472	-	-	-	-	-	-
	Index	**0.5629**	0.5579	0.5679	-	-	-	-	-	-
7-char. ATC	Index Sex Age	**0.5707**	0.5659	0.5756	-	-	-	-	-	-
	Sex Age	**0.5424**	0.5377	0.5472	-	-	-	-	-	-
	Index	**0.5631**	0.5581	0.5681	-	-	-	-	-	-

Table 10.

Malignant melanoma of skin (ICD-10: C43)

Index, Model, Year of Event 2015-2019		Look-back period								
		2014			2012-2014			2010-2014		
		AUROC	95% LCL	95% UCL	AUROC	95% LCL	95% UCL	AUROC	95% LCL	95% UCL
Nordic Multimorbidity Index	Index Sex Age	**0.5661**	0.5596	0.5725	**0.5668**	0.5603	0.5732	**0.5668**	0.5603	0.5732
	Sex Age	**0.5616**	0.5551	0.5682	**0.5616**	0.5551	0.5682	**0.5616**	0.5551	0.5682
	Index	**0.5179**	0.5115	0.5243	**0.5172**	0.5108	0.5236	**0.5154**	0.5090	0.5218
Charlson Comorbidity Index	Index Sex Age	**0.5618**	0.5552	0.5683	**0.5619**	0.5554	0.5684	**0.5620**	0.5555	0.5686
	Sex Age	**0.5616**	0.5551	0.5682	**0.5616**	0.5551	0.5682	**0.5616**	0.5551	0.5682
	Index	**0.5046**	0.5005	0.5087	**0.5040**	0.4991	0.5089	**0.5071**	0.5019	0.5124
Elixhauser Comorbidity Index	Index Sex Age	**0.5618**	0.5553	0.5683	**0.5616**	0.5551	0.5681	**0.5615**	0.5549	0.5680
	Sex Age	**0.5616**	0.5551	0.5682	**0.5616**	0.5551	0.5682	**0.5616**	0.5551	0.5682
	Index	**0.5097**	0.5055	0.5138	**0.5112**	0.5064	0.5161	**0.5129**	0.5077	0.5180
3-char. ICD-10 main diag.	Index Sex Age	**0.5621**	0.5556	0.5686	**0.5623**	0.5558	0.5688	**0.5621**	0.5556	0.5686
	Sex Age	**0.5616**	0.5551	0.5682	**0.5616**	0.5551	0.5682	**0.5616**	0.5551	0.5682
	Index	**0.4998**	0.4959	0.5036	**0.4972**	0.4920	0.5024	**0.4937**	0.4880	0.4994
Visits to inpatient care	Index Sex Age	**0.5624**	0.5559	0.5689	**0.5623**	0.5558	0.5688	**0.5623**	0.5558	0.5688
	Sex Age	**0.5616**	0.5551	0.5682	**0.5616**	0.5551	0.5682	**0.5616**	0.5551	0.5682
	Index	**0.5002**	0.4963	0.5040	**0.4973**	0.4921	0.5025	**0.4943**	0.4886	0.5000
Days in inpatient care	Index Sex Age	**0.5632**	0.5567	0.5697	**0.5640**	0.5576	0.5705	**0.5635**	0.5570	0.5700
	Sex Age	**0.5616**	0.5551	0.5682	**0.5616**	0.5551	0.5682	**0.5616**	0.5551	0.5682
	Index	**0.5003**	0.4964	0.5042	**0.4986**	0.4934	0.5038	**0.4955**	0.4898	0.5012
1-char. ATC	Index Sex Age	**0.5631**	0.5566	0.5697	-	-	-	-	-	-
	Sex Age	**0.5616**	0.5551	0.5682	-	-	-	-	-	-
	Index	**0.5151**	0.5089	0.5214	-	-	-	-	-	-
3-char. ATC	Index Sex Age	**0.5622**	0.5557	0.5688	-	-	-	-	-	-
	Sex Age	**0.5616**	0.5551	0.5682	-	-	-	-	-	-
	Index	**0.5149**	0.5086	0.5211	-	-	-	-	-	-
4-char. ATC	Index Sex Age	**0.5620**	0.5555	0.5685	-	-	-	-	-	-
	Sex Age	**0.5616**	0.5551	0.5682	-	-	-	-	-	-
	Index	**0.5128**	0.5066	0.5190	-	-	-	-	-	-
5-char. ATC	Index Sex Age	**0.5620**	0.5555	0.5685	-	-	-	-	-	-
	Sex Age	**0.5616**	0.5551	0.5682	-	-	-	-	-	-
	Index	**0.5131**	0.5069	0.5194	-	-	-	-	-	-
7-char. ATC	Index Sex Age	**0.5620**	0.5555	0.5685	-	-	-	-	-	-
	Sex Age	**0.5616**	0.5551	0.5682	-	-	-	-	-	-
	Index	**0.5132**	0.5070	0.5194	-	-	-	-	-	-

Table 11.

Breast cancer (ICD-10: C50)

Index, Model, Year of Event 2015-2019		Look-back period								
		2014			2012-2014			2010-2014		
		AUROC	95% LCL	95% UCL	AUROC	95% LCL	95% UCL	AUROC	95% LCL	95% UCL
Nordic Multimorbidity Index	Index Sex Age	**0.5333**	0.5286	0.5379	**0.5330**	0.5284	0.5377	**0.5332**	0.5286	0.5379
	Sex Age	**0.5316**	0.5270	0.5362	**0.5316**	0.5270	0.5362	**0.5316**	0.5270	0.5362
	Index	**0.5068**	0.5022	0.5115	**0.5065**	0.5019	0.5112	**0.5058**	0.5011	0.5104
Charlson Comorbidity Index	Index Sex Age	**0.5343**	0.5296	0.5389	**0.5343**	0.5297	0.5390	**0.5332**	0.5286	0.5379
	Sex Age	**0.5316**	0.5270	0.5362	**0.5316**	0.5270	0.5362	**0.5316**	0.5270	0.5362
	Index	**0.5086**	0.5060	0.5111	**0.5113**	0.5081	0.5144	**0.5099**	0.5064	0.5133
Elixhauser Comorbidity Index	Index Sex Age	**0.5331**	0.5285	0.5378	**0.5324**	0.5277	0.5370	**0.5323**	0.5276	0.5369
	Sex Age	**0.5316**	0.5270	0.5362	**0.5316**	0.5270	0.5362	**0.5316**	0.5270	0.5362
	Index	**0.5038**	0.5012	0.5063	**0.5031**	0.4999	0.5062	**0.5018**	0.4984	0.5052
3-char. ICD-10 main diag.	Index Sex Age	**0.5339**	0.5293	0.5385	**0.5333**	0.5287	0.5380	**0.5327**	0.5280	0.5373
	Sex Age	**0.5316**	0.5270	0.5362	**0.5316**	0.5270	0.5362	**0.5316**	0.5270	0.5362
	Index	**0.5080**	0.5054	0.5106	**0.5090**	0.5054	0.5126	**0.5074**	0.5033	0.5114
Visits to inpatient care	Index Sex Age	**0.5342**	0.5296	0.5389	**0.5335**	0.5288	0.5381	**0.5327**	0.5281	0.5374
	Sex Age	**0.5316**	0.5270	0.5362	**0.5316**	0.5270	0.5362	**0.5316**	0.5270	0.5362
	Index	**0.5082**	0.5056	0.5108	**0.5089**	0.5053	0.5126	**0.5074**	0.5034	0.5115
Days in inpatient care	Index Sex Age	**0.5344**	0.5298	0.5390	**0.5342**	0.5295	0.5388	**0.5334**	0.5288	0.5381
	Sex Age	**0.5316**	0.5270	0.5362	**0.5316**	0.5270	0.5362	**0.5316**	0.5270	0.5362
	Index	**0.5084**	0.5058	0.5110	**0.5098**	0.5062	0.5135	**0.5089**	0.5048	0.5129
1-char. ATC	Index Sex Age	**0.5331**	0.5285	0.5378	-	-	-	-	-	-
	Sex Age	**0.5316**	0.5270	0.5362	-	-	-	-	-	-
	Index	**0.5031**	0.4984	0.5078	-	-	-	-	-	-
3-char. ATC	Index Sex Age	**0.5321**	0.5274	0.5367	-	-	-	-	-	-
	Sex Age	**0.5316**	0.5270	0.5362	-	-	-	-	-	-
	Index	**0.4989**	0.4942	0.5037	-	-	-	-	-	-
4-char. ATC	Index Sex Age	**0.5321**	0.5274	0.5367	-	-	-	-	-	-
	Sex Age	**0.5316**	0.5270	0.5362	-	-	-	-	-	-
	Index	**0.4985**	0.4938	0.5033	-	-	-	-	-	-
5-char. ATC	Index Sex Age	**0.5321**	0.5274	0.5367	-	-	-	-	-	-
	Sex Age	**0.5316**	0.5270	0.5362	-	-	-	-	-	-
	Index	**0.4991**	0.4944	0.5038	-	-	-	-	-	-
7-char. ATC	Index Sex Age	**0.5320**	0.5274	0.5367	-	-	-	-	-	-
	Sex Age	**0.5316**	0.5270	0.5362	-	-	-	-	-	-
	Index	**0.4992**	0.4945	0.5039	-	-	-	-	-	-

Table 12.

Prostate cancer (ICD-10: C61)

Index, Model, Year of Event 2015-2019		Look-back period								
		2014			2012-2014			2010-2014		
		AUROC	95% LCL	95% UCL	AUROC	95% LCL	95% UCL	AUROC	95% LCL	95% UCL
Nordic Multimorbidity Index	Index Sex Age	**0.5414**	0.5382	0.5446	**0.5440**	0.5408	0.5472	**0.5458**	0.5426	0.5490
	Sex Age	**0.5082**	0.5050	0.5115	**0.5082**	0.5050	0.5115	**0.5082**	0.5050	0.5115
	Index	**0.5349**	0.5318	0.5380	**0.5370**	0.5339	0.5401	**0.5389**	0.5358	0.5420
Charlson Comorbidity Index	Index Sex Age	**0.5294**	0.5262	0.5326	**0.5355**	0.5322	0.5387	**0.5379**	0.5346	0.5411
	Sex Age	**0.5082**	0.5050	0.5115	**0.5082**	0.5050	0.5115	**0.5082**	0.5050	0.5115
	Index	**0.5196**	0.5178	0.5214	**0.5253**	0.5231	0.5276	**0.5281**	0.5257	0.5306
Elixhauser Comorbidity Index	Index Sex Age	**0.5265**	0.5232	0.5297	**0.5312**	0.5279	0.5344	**0.5336**	0.5304	0.5369
	Sex Age	**0.5082**	0.5050	0.5115	**0.5082**	0.5050	0.5115	**0.5082**	0.5050	0.5115
	Index	**0.5166**	0.5147	0.5185	**0.5215**	0.5193	0.5237	**0.5244**	0.5220	0.5268
3-char. ICD-10 main diag.	Index Sex Age	**0.5218**	0.5186	0.5250	**0.5264**	0.5231	0.5297	**0.5285**	0.5252	0.5317
	Sex Age	**0.5082**	0.5050	0.5115	**0.5082**	0.5050	0.5115	**0.5082**	0.5050	0.5115
	Index	**0.5127**	0.5109	0.5146	**0.5182**	0.5156	0.5208	**0.5219**	0.5191	0.5247
Visits to inpatient care	Index Sex Age	**0.5219**	0.5186	0.5251	**0.5262**	0.5230	0.5295	**0.5288**	0.5255	0.5320
	Sex Age	**0.5082**	0.5050	0.5115	**0.5082**	0.5050	0.5115	**0.5082**	0.5050	0.5115
	Index	**0.5128**	0.5109	0.5147	**0.5186**	0.5160	0.5211	**0.5221**	0.5193	0.5249
Days in inpatient care	Index Sex Age	**0.5224**	0.5192	0.5257	**0.5286**	0.5254	0.5319	**0.5316**	0.5284	0.5349
	Sex Age	**0.5082**	0.5050	0.5115	**0.5082**	0.5050	0.5115	**0.5082**	0.5050	0.5115
	Index	**0.5131**	0.5112	0.5150	**0.5195**	0.5169	0.5220	**0.5234**	0.5206	0.5262
1-char. ATC	Index Sex Age	**0.5175**	0.5142	0.5208	-	-	-	-	-	-
	Sex Age	**0.5082**	0.5050	0.5115	-	-	-	-	-	-
	Index	**0.5144**	0.5112	0.5177	-	-	-	-	-	-
3-char. ATC	Index Sex Age	**0.5238**	0.5205	0.5271	-	-	-	-	-	-
	Sex Age	**0.5082**	0.5050	0.5115	-	-	-	-	-	-
	Index	**0.5197**	0.5165	0.5229	-	-	-	-	-	-
4-char. ATC	Index Sex Age	**0.5249**	0.5217	0.5282	-	-	-	-	-	-
	Sex Age	**0.5082**	0.5050	0.5115	-	-	-	-	-	-
	Index	**0.5206**	0.5174	0.5238	-	-	-	-	-	-
5-char. ATC	Index Sex Age	**0.5259**	0.5227	0.5292	-	-	-	-	-	-
	Sex Age	**0.5082**	0.5050	0.5115	-	-	-	-	-	-
	Index	**0.5214**	0.5182	0.5246	-	-	-	-	-	-
7-char. ATC	Index Sex Age	**0.5261**	0.5228	0.5293	-	-	-	-	-	-
	Sex Age	**0.5082**	0.5050	0.5115	-	-	-	-	-	-
	Index	**0.5215**	0.5183	0.5247	-	-	-	-	-	-

Tables 13 to 18 are for the age group 80-plus years of age.

Table 13.

Carcinoma, all (ICD-10: C00-C97)

Index, Model, Year of Event 2015-2019		Look-back period								
		2014			2012-2014			2010-2014		
		AUROC	95% LCL	95% UCL	AUROC	95% LCL	95% UCL	AUROC	95% LCL	95% UCL
Nordic Multimorbidity Index	Index Sex Age	**0.6150**	0.6123	0.6178	**0.6167**	0.6140	0.6194	**0.6173**	0.6146	0.6200
	Sex Age	**0.5983**	0.5955	0.6010	**0.5983**	0.5955	0.6010	**0.5983**	0.5955	0.6010
	Index	**0.5716**	0.5688	0.5743	**0.5730**	0.5703	0.5757	**0.5737**	0.5710	0.5764
Charlson Comorbidity Index	Index Sex Age	**0.6022**	0.5995	0.6050	**0.6045**	0.6018	0.6073	**0.6049**	0.6021	0.6076
	Sex Age	**0.5983**	0.5955	0.6010	**0.5983**	0.5955	0.6010	**0.5983**	0.5955	0.6010
	Index	**0.5172**	0.5153	0.5191	**0.5289**	0.5266	0.5313	**0.5326**	0.5301	0.5351
Elixhauser Comorbidity Index	Index Sex Age	**0.6010**	0.5982	0.6037	**0.6023**	0.5995	0.6051	**0.6024**	0.5996	0.6052
	Sex Age	**0.5983**	0.5955	0.6010	**0.5983**	0.5955	0.6010	**0.5983**	0.5955	0.6010
	Index	**0.5106**	0.5086	0.5125	**0.5196**	0.5173	0.5219	**0.5214**	0.5189	0.5238
3-char. ICD-10 main diag.	Index Sex Age	**0.6018**	0.5991	0.6046	**0.6041**	0.6014	0.6069	**0.6052**	0.6025	0.6080
	Sex Age	**0.5983**	0.5955	0.6010	**0.5983**	0.5955	0.6010	**0.5983**	0.5955	0.6010
	Index	**0.5214**	0.5193	0.5234	**0.5360**	0.5335	0.5385	**0.5412**	0.5386	0.5439
Visits to inpatient care	Index Sex Age	**0.6019**	0.5991	0.6047	**0.6041**	0.6013	0.6068	**0.6048**	0.6021	0.6076
	Sex Age	**0.5983**	0.5955	0.6010	**0.5983**	0.5955	0.6010	**0.5983**	0.5955	0.6010
	Index	**0.5216**	0.5196	0.5237	**0.5361**	0.5336	0.5387	**0.5411**	0.5384	0.5438
Days in inpatient care	Index Sex Age	**0.6032**	0.6005	0.6060	**0.6069**	0.6042	0.6097	**0.6090**	0.6063	0.6118
	Sex Age	**0.5983**	0.5955	0.6010	**0.5983**	0.5955	0.6010	**0.5983**	0.5955	0.6010
	Index	**0.5236**	0.5216	0.5256	**0.5428**	0.5403	0.5453	**0.5510**	0.5483	0.5536
1-char. ATC	Index Sex Age	**0.5983**	0.5956	0.6011	-	-	-	-	-	-
	Sex Age	**0.5983**	0.5955	0.6010	-	-	-	-	-	-
	Index	**0.5147**	0.5118	0.5176	-	-	-	-	-	-
3-char. ATC	Index Sex Age	**0.5988**	0.5960	0.6016	-	-	-	-	-	-
	Sex Age	**0.5983**	0.5955	0.6010	-	-	-	-	-	-
	Index	**0.5218**	0.5189	0.5247	-	-	-	-	-	-
4-char. ATC	Index Sex Age	**0.5992**	0.5964	0.6019	-	-	-	-	-	-
	Sex Age	**0.5983**	0.5955	0.6010	-	-	-	-	-	-
	Index	**0.5249**	0.5221	0.5278	-	-	-	-	-	-
5-char. ATC	Index Sex Age	**0.5994**	0.5966	0.6022	-	-	-	-	-	-
	Sex Age	**0.5983**	0.5955	0.6010	-	-	-	-	-	-
	Index	**0.5258**	0.5230	0.5287	-	-	-	-	-	-
7-char. ATC	Index Sex Age	**0.5994**	0.5966	0.6022	-	-	-	-	-	-
	Sex Age	**0.5983**	0.5955	0.6010	-	-	-	-	-	-
	Index	**0.5258**	0.5230	0.5287	-	-	-	-	-	-

Table 14.

Colorectal cancer (ICD-10: C18-C21)

Index, Model, Year of Event 2015-2019		Look-back period								
		2014			2012-2014			2010-2014		
		AUROC	95% LCL	95% UCL	AUROC	95% LCL	95% UCL	AUROC	95% LCL	95% UCL
Nordic Multimorbidity Index	Index Sex Age	**0.6149**	0.6089	0.6209	**0.6176**	0.6117	0.6236	**0.6183**	0.6124	0.6242
	Sex Age	**0.5921**	0.5860	0.5982	**0.5921**	0.5860	0.5982	**0.5921**	0.5860	0.5982
	Index	**0.5810**	0.5747	0.5872	**0.5845**	0.5783	0.5907	**0.5857**	0.5794	0.5919
Charlson Comorbidity Index	Index Sex Age	**0.5984**	0.5923	0.6045	**0.6028**	0.5968	0.6089	**0.6037**	0.5976	0.6098
	Sex Age	**0.5921**	0.5860	0.5982	**0.5921**	0.5860	0.5982	**0.5921**	0.5860	0.5982
	Index	**0.5192**	0.5145	0.5238	**0.5361**	0.5306	0.5416	**0.5407**	0.5348	0.5465
Elixhauser Comorbidity Index	Index Sex Age	**0.5967**	0.5906	0.6028	**0.5998**	0.5937	0.6059	**0.6000**	0.5939	0.6061
	Sex Age	**0.5921**	0.5860	0.5982	**0.5921**	0.5860	0.5982	**0.5921**	0.5860	0.5982
	Index	**0.5157**	0.5110	0.5203	**0.5295**	0.5241	0.5350	**0.5323**	0.5266	0.5381
3-char. ICD-10 main diag.	Index Sex Age	**0.5979**	0.5918	0.6040	**0.6030**	0.5969	0.6090	**0.6051**	0.5990	0.6111
	Sex Age	**0.5921**	0.5860	0.5982	**0.5921**	0.5860	0.5982	**0.5921**	0.5860	0.5982
	Index	**0.5270**	0.5223	0.5317	**0.5484**	0.5426	0.5542	**0.5547**	0.5485	0.5608
Visits to inpatient care	Index Sex Age	**0.5981**	0.5920	0.6042	**0.6031**	0.5971	0.6092	**0.6042**	0.5981	0.6103
	Sex Age	**0.5921**	0.5860	0.5982	**0.5921**	0.5860	0.5982	**0.5921**	0.5860	0.5982
	Index	**0.5274**	0.5227	0.5321	**0.5494**	0.5436	0.5553	**0.5553**	0.5491	0.5615
Days in inpatient care	Index Sex Age	**0.5993**	0.5932	0.6054	**0.6045**	0.5984	0.6105	**0.6073**	0.6012	0.6133
	Sex Age	**0.5921**	0.5860	0.5982	**0.5921**	0.5860	0.5982	**0.5921**	0.5860	0.5982
	Index	**0.5295**	0.5249	0.5342	**0.5556**	0.5499	0.5613	**0.5647**	0.5587	0.5708
1-char. ATC	Index Sex Age	**0.5971**	0.5909	0.6032	-	-	-	-	-	-
	Sex Age	**0.5921**	0.5860	0.5982	-	-	-	-	-	-
	Index	**0.5368**	0.5302	0.5435	-	-	-	-	-	-
3-char. ATC	Index Sex Age	**0.5997**	0.5936	0.6058	-	-	-	-	-	-
	Sex Age	**0.5921**	0.5860	0.5982	-	-	-	-	-	-
	Index	**0.5434**	0.5368	0.5500	-	-	-	-	-	-
4-char. ATC	Index Sex Age	**0.6004**	0.5943	0.6065	-	-	-	-	-	-
	Sex Age	**0.5921**	0.5860	0.5982	-	-	-	-	-	-
	Index	**0.5459**	0.5393	0.5524	-	-	-	-	-	-
5-char. ATC	Index Sex Age	**0.6012**	0.5951	0.6073	-	-	-	-	-	-
	Sex Age	**0.5921**	0.5860	0.5982	-	-	-	-	-	-
	Index	**0.5472**	0.5406	0.5538	-	-	-	-	-	-
7-char. ATC	Index Sex Age	**0.6013**	0.5952	0.6074	-	-	-	-	-	-
	Sex Age	**0.5921**	0.5860	0.5982	-	-	-	-	-	-
	Index	**0.5474**	0.5409	0.5540	-	-	-	-	-	-

Table 15.

Lung cancer (ICD-10: C34)

Index, Model, Year of Event 2015-2019		Look-back period								
		2014			2012-2014			2010-2014		
		AUROC	95% LCL	95% UCL	AUROC	95% LCL	95% UCL	AUROC	95% LCL	95% UCL
Nordic Multimorbidity Index	Index Sex Age	**0.6701**	0.6616	0.6787	**0.6698**	0.6612	0.6784	**0.6694**	0.6609	0.6780
	Sex Age	**0.6673**	0.6586	0.6760	**0.6673**	0.6586	0.6760	**0.6673**	0.6586	0.6760
	Index	**0.5534**	0.5436	0.5632	**0.5493**	0.5395	0.5591	**0.5473**	0.5375	0.5572
Charlson Comorbidity Index	Index Sex Age	**0.6678**	0.6592	0.6765	**0.6677**	0.6591	0.6764	**0.6671**	0.6584	0.6758
	Sex Age	**0.6673**	0.6586	0.6760	**0.6673**	0.6586	0.6760	**0.6673**	0.6586	0.6760
	Index	**0.5018**	0.4941	0.5095	**0.5053**	0.4963	0.5144	**0.5001**	0.4906	0.5096
Elixhauser Comorbidity Index	Index Sex Age	**0.6682**	0.6595	0.6768	**0.6688**	0.6602	0.6774	**0.6678**	0.6591	0.6764
	Sex Age	**0.6673**	0.6586	0.6760	**0.6673**	0.6586	0.6760	**0.6673**	0.6586	0.6760
	Index	**0.5059**	0.4985	0.5134	**0.5128**	0.5042	0.5213	**0.5083**	0.4993	0.5173
3-char. ICD-10 main diag.	Index Sex Age	**0.6692**	0.6605	0.6778	**0.6699**	0.6613	0.6785	**0.6699**	0.6612	0.6785
	Sex Age	**0.6673**	0.6586	0.6760	**0.6673**	0.6586	0.6760	**0.6673**	0.6586	0.6760
	Index	**0.5260**	0.5187	0.5334	**0.5366**	0.5275	0.5458	**0.5403**	0.5308	0.5499
Visits to inpatient care	Index Sex Age	**0.6691**	0.6605	0.6778	**0.6700**	0.6613	0.6786	**0.6698**	0.6611	0.6784
	Sex Age	**0.6673**	0.6586	0.6760	**0.6673**	0.6586	0.6760	**0.6673**	0.6586	0.6760
	Index	**0.5259**	0.5185	0.5333	**0.5361**	0.5269	0.5454	**0.5393**	0.5296	0.5490
Days in inpatient care	Index Sex Age	**0.6694**	0.6607	0.6780	**0.6705**	0.6619	0.6791	**0.6707**	0.6621	0.6793
	Sex Age	**0.6673**	0.6586	0.6760	**0.6673**	0.6586	0.6760	**0.6673**	0.6586	0.6760
	Index	**0.5279**	0.5206	0.5353	**0.5432**	0.5341	0.5522	**0.5489**	0.5394	0.5583
1-char. ATC	Index Sex Age	**0.6687**	0.6600	0.6774	-	-	-	-	-	-
	Sex Age	**0.6673**	0.6586	0.6760	-	-	-	-	-	-
	Index	**0.5015**	0.4910	0.5119	-	-	-	-	-	-
3-char. ATC	Index Sex Age	**0.6682**	0.6595	0.6769	-	-	-	-	-	-
	Sex Age	**0.6673**	0.6586	0.6760	-	-	-	-	-	-
	Index	**0.5074**	0.4970	0.5178	-	-	-	-	-	-
4-char. ATC	Index Sex Age	**0.6683**	0.6596	0.6770	-	-	-	-	-	-
	Sex Age	**0.6673**	0.6586	0.6760	-	-	-	-	-	-
	Index	**0.5060**	0.4957	0.5164	-	-	-	-	-	-
5-char. ATC	Index Sex Age	**0.6682**	0.6595	0.6769	-	-	-	-	-	-
	Sex Age	**0.6673**	0.6586	0.6760	-	-	-	-	-	-
	Index	**0.5075**	0.4971	0.5178	-	-	-	-	-	-
7-char. ATC	Index Sex Age	**0.6682**	0.6595	0.6769	-	-	-	-	-	-
	Sex Age	**0.6673**	0.6586	0.6760	-	-	-	-	-	-
	Index	**0.5067**	0.4963	0.5171	-	-	-	-	-	-

Table 16.

Malignant melanoma of skin (ICD-10: C43)

Index, Model, Year of Event 2015-2019		Look-back period								
		2014			2012-2014			2010-2014		
		AUROC	95% LCL	95% UCL	AUROC	95% LCL	95% UCL	AUROC	95% LCL	95% UCL
Nordic Multimorbidity Index	Index Sex Age	**0.6046**	0.5942	0.6149	**0.6055**	0.5951	0.6159	**0.6057**	0.5953	0.6161
	Sex Age	**0.5862**	0.5755	0.5969	**0.5862**	0.5755	0.5969	**0.5862**	0.5755	0.5969
	Index	**0.5645**	0.5542	0.5748	**0.5643**	0.5540	0.5746	**0.5631**	0.5528	0.5734
Charlson Comorbidity Index	Index Sex Age	**0.5921**	0.5815	0.6027	**0.5930**	0.5823	0.6037	**0.5929**	0.5822	0.6035
	Sex Age	**0.5862**	0.5755	0.5969	**0.5862**	0.5755	0.5969	**0.5862**	0.5755	0.5969
	Index	**0.5142**	0.5064	0.5219	**0.5197**	0.5104	0.5290	**0.5193**	0.5094	0.5291
Elixhauser Comorbidity Index	Index Sex Age	**0.5889**	0.5783	0.5996	**0.5883**	0.5776	0.5990	**0.5886**	0.5779	0.5992
	Sex Age	**0.5862**	0.5755	0.5969	**0.5862**	0.5755	0.5969	**0.5862**	0.5755	0.5969
	Index	**0.5063**	0.4984	0.5142	**0.5072**	0.4979	0.5164	**0.5087**	0.4990	0.5184
3-char. ICD-10 main diag.	Index Sex Age	**0.5930**	0.5824	0.6036	**0.5934**	0.5828	0.6040	**0.5956**	0.5850	0.6062
	Sex Age	**0.5862**	0.5755	0.5969	**0.5862**	0.5755	0.5969	**0.5862**	0.5755	0.5969
	Index	**0.5253**	0.5176	0.5331	**0.5345**	0.5247	0.5442	**0.5403**	0.5301	0.5504
Visits to inpatient care	Index Sex Age	**0.5935**	0.5829	0.6041	**0.5929**	0.5823	0.6036	**0.5949**	0.5843	0.6056
	Sex Age	**0.5862**	0.5755	0.5969	**0.5862**	0.5755	0.5969	**0.5862**	0.5755	0.5969
	Index	**0.5262**	0.5185	0.5339	**0.5352**	0.5254	0.5450	**0.5420**	0.5318	0.5522
Days in inpatient care	Index Sex Age	**0.5924**	0.5818	0.6030	**0.5928**	0.5821	0.6034	**0.5963**	0.5856	0.6069
	Sex Age	**0.5862**	0.5755	0.5969	**0.5862**	0.5755	0.5969	**0.5862**	0.5755	0.5969
	Index	**0.5267**	0.5189	0.5344	**0.5387**	0.5290	0.5484	**0.5477**	0.5375	0.5578
1-char. ATC	Index Sex Age	**0.5864**	0.5758	0.5971	-	-	-	-	-	-
	Sex Age	**0.5862**	0.5755	0.5969	-	-	-	-	-	-
	Index	**0.5065**	0.4957	0.5172	-	-	-	-	-	-
3-char. ATC	Index Sex Age	**0.5864**	0.5757	0.5971	-	-	-	-	-	-
	Sex Age	**0.5862**	0.5755	0.5969	-	-	-	-	-	-
	Index	**0.5134**	0.5028	0.5241	-	-	-	-	-	-
4-char. ATC	Index Sex Age	**0.5868**	0.5762	0.5975	-	-	-	-	-	-
	Sex Age	**0.5862**	0.5755	0.5969	-	-	-	-	-	-
	Index	**0.5176**	0.5070	0.5281	-	-	-	-	-	-
5-char. ATC	Index Sex Age	**0.5868**	0.5762	0.5975	-	-	-	-	-	-
	Sex Age	**0.5862**	0.5755	0.5969	-	-	-	-	-	-
	Index	**0.5170**	0.5064	0.5276	-	-	-	-	-	-
7-char. ATC	Index Sex Age	**0.5868**	0.5761	0.5974	-	-	-	-	-	-
	Sex Age	**0.5862**	0.5755	0.5969	-	-	-	-	-	-
	Index	**0.5165**	0.5059	0.5271	-	-	-	-	-	-

Table 17.

Breast cancer (ICD-10: C50)

Index, Model, Year of Event 2015-2019		Look-back period								
		2014			2012-2014			2010-2014		
		AUROC	95% LCL	95% UCL	AUROC	95% LCL	95% UCL	AUROC	95% LCL	95% UCL
Nordic Multimorbidity Index	Index Sex Age	**0.5806**	0.5729	0.5883	**0.5824**	0.5748	0.5900	**0.5838**	0.5762	0.5914
	Sex Age	**0.5672**	0.5594	0.5750	**0.5672**	0.5594	0.5750	**0.5672**	0.5594	0.5750
	Index	**0.5520**	0.5441	0.5599	**0.5545**	0.5467	0.5624	**0.5575**	0.5497	0.5653
Charlson Comorbidity Index	Index Sex Age	**0.5719**	0.5641	0.5797	**0.5728**	0.5650	0.5805	**0.5737**	0.5659	0.5815
	Sex Age	**0.5672**	0.5594	0.5750	**0.5672**	0.5594	0.5750	**0.5672**	0.5594	0.5750
	Index	**0.5174**	0.5120	0.5228	**0.5241**	0.5173	0.5309	**0.5292**	0.5220	0.5364
Elixhauser Comorbidity Index	Index Sex Age	**0.5690**	0.5612	0.5767	**0.5692**	0.5614	0.5770	**0.5690**	0.5612	0.5768
	Sex Age	**0.5672**	0.5594	0.5750	**0.5672**	0.5594	0.5750	**0.5672**	0.5594	0.5750
	Index	**0.5091**	0.5035	0.5146	**0.5108**	0.5041	0.5175	**0.5126**	0.5055	0.5197
3-char. ICD-10 main diag.	Index Sex Age	**0.5709**	0.5631	0.5787	**0.5716**	0.5639	0.5794	**0.5733**	0.5655	0.5810
	Sex Age	**0.5672**	0.5594	0.5750	**0.5672**	0.5594	0.5750	**0.5672**	0.5594	0.5750
	Index	**0.5167**	0.5107	0.5227	**0.5253**	0.5179	0.5326	**0.5321**	0.5244	0.5399
Visits to inpatient care	Index Sex Age	**0.5707**	0.5629	0.5785	**0.5716**	0.5638	0.5794	**0.5735**	0.5657	0.5813
	Sex Age	**0.5672**	0.5594	0.5750	**0.5672**	0.5594	0.5750	**0.5672**	0.5594	0.5750
	Index	**0.5167**	0.5107	0.5227	**0.5247**	0.5172	0.5322	**0.5315**	0.5238	0.5393
Days in inpatient care	Index Sex Age	**0.5721**	0.5643	0.5798	**0.5735**	0.5658	0.5813	**0.5765**	0.5687	0.5842
	Sex Age	**0.5672**	0.5594	0.5750	**0.5672**	0.5594	0.5750	**0.5672**	0.5594	0.5750
	Index	**0.5182**	0.5122	0.5242	**0.5297**	0.5223	0.5370	**0.5393**	0.5316	0.5470
1-char. ATC	Index Sex Age	**0.5675**	0.5597	0.5754	-	-	-	-	-	-
	Sex Age	**0.5672**	0.5594	0.5750	-	-	-	-	-	-
	Index	**0.5103**	0.5020	0.5186	-	-	-	-	-	-
3-char. ATC	Index Sex Age	**0.5684**	0.5605	0.5762	-	-	-	-	-	-
	Sex Age	**0.5672**	0.5594	0.5750	-	-	-	-	-	-
	Index	**0.5150**	0.5068	0.5233	-	-	-	-	-	-
4-char. ATC	Index Sex Age	**0.5693**	0.5615	0.5771	-	-	-	-	-	-
	Sex Age	**0.5672**	0.5594	0.5750	-	-	-	-	-	-
	Index	**0.5186**	0.5104	0.5269	-	-	-	-	-	-
5-char. ATC	Index Sex Age	**0.5698**	0.5620	0.5776	-	-	-	-	-	-
	Sex Age	**0.5672**	0.5594	0.5750	-	-	-	-	-	-
	Index	**0.5205**	0.5122	0.5287	-	-	-	-	-	-
7-char. ATC	Index Sex Age	**0.5699**	0.5621	0.5777	-	-	-	-	-	-
	Sex Age	**0.5672**	0.5594	0.5750	-	-	-	-	-	-
	Index	**0.5207**	0.5125	0.5289	-	-	-	-	-	-

Table 18.

Prostate cancer (ICD-10: C61)

Index, Model, Year of Event 2015-2019		Look-back period								
		2014			2012-2014			2010-2014		
		AUROC	95% LCL	95% UCL	AUROC	95% LCL	95% UCL	AUROC	95% LCL	95% UCL
Nordic Multimorbidity Index	Index Sex Age	**0.6484**	0.6419	0.6549	**0.6517**	0.6452	0.6582	**0.6532**	0.6467	0.6597
	Sex Age	**0.6044**	0.5977	0.6112	**0.6044**	0.5977	0.6112	**0.6044**	0.5977	0.6112
	Index	**0.6148**	0.6080	0.6215	**0.6202**	0.6135	0.6269	**0.6227**	0.6159	0.6294
Charlson Comorbidity Index	Index Sex Age	**0.6229**	0.6162	0.6296	**0.6301**	0.6234	0.6368	**0.6309**	0.6242	0.6376
	Sex Age	**0.6044**	0.5977	0.6112	**0.6044**	0.5977	0.6112	**0.6044**	0.5977	0.6112
	Index	**0.5486**	0.5436	0.5536	**0.5706**	0.5645	0.5767	**0.5772**	0.5707	0.5837
Elixhauser Comorbidity Index	Index Sex Age	**0.6224**	0.6157	0.6291	**0.6284**	0.6217	0.6351	**0.6293**	0.6225	0.6360
	Sex Age	**0.6044**	0.5977	0.6112	**0.6044**	0.5977	0.6112	**0.6044**	0.5977	0.6112
	Index	**0.5468**	0.5417	0.5519	**0.5676**	0.5615	0.5736	**0.5723**	0.5660	0.5787
3-char. ICD-10 main diag.	Index Sex Age	**0.6167**	0.6100	0.6235	**0.6250**	0.6183	0.6318	**0.6287**	0.6219	0.6354
	Sex Age	**0.6044**	0.5977	0.6112	**0.6044**	0.5977	0.6112	**0.6044**	0.5977	0.6112
	Index	**0.5418**	0.5366	0.5470	**0.5684**	0.5619	0.5749	**0.5773**	0.5705	0.5841
Visits to inpatient care	Index Sex Age	**0.6167**	0.6099	0.6234	**0.6257**	0.6189	0.6324	**0.6285**	0.6218	0.6353
	Sex Age	**0.6044**	0.5977	0.6112	**0.6044**	0.5977	0.6112	**0.6044**	0.5977	0.6112
	Index	**0.5424**	0.5373	0.5476	**0.5686**	0.5621	0.5751	**0.5762**	0.5694	0.5831
Days in inpatient care	Index Sex Age	**0.6193**	0.6126	0.6260	**0.6304**	0.6237	0.6371	**0.6346**	0.6280	0.6413
	Sex Age	**0.6044**	0.5977	0.6112	**0.6044**	0.5977	0.6112	**0.6044**	0.5977	0.6112
	Index	**0.5447**	0.5396	0.5498	**0.5759**	0.5695	0.5822	**0.5865**	0.5798	0.5931
1-char. ATC	Index Sex Age	**0.6208**	0.6140	0.6277	-	-	-	-	-	-
	Sex Age	**0.6044**	0.5977	0.6112	-	-	-	-	-	-
	Index	**0.5646**	0.5570	0.5722	-	-	-	-	-	-
3-char. ATC	Index Sex Age	**0.6258**	0.6190	0.6327	-	-	-	-	-	-
	Sex Age	**0.6044**	0.5977	0.6112	-	-	-	-	-	-
	Index	**0.5723**	0.5648	0.5797	-	-	-	-	-	-
4-char. ATC	Index Sex Age	**0.6279**	0.6211	0.6347	-	-	-	-	-	-
	Sex Age	**0.6044**	0.5977	0.6112	-	-	-	-	-	-
	Index	**0.5759**	0.5685	0.5833	-	-	-	-	-	-
5-char. ATC	Index Sex Age	**0.6280**	0.6212	0.6348	-	-	-	-	-	-
	Sex Age	**0.6044**	0.5977	0.6112	-	-	-	-	-	-
	Index	**0.5765**	0.5692	0.5839	-	-	-	-	-	-
7-char. ATC	Index Sex Age	**0.6283**	0.6215	0.6351	-	-	-	-	-	-
	Sex Age	**0.6044**	0.5977	0.6112	-	-	-	-	-	-
	Index	**0.5765**	0.5691	0.5838	-	-	-	-	-	-

Figure 3.

In the two panels below, the Area under the Receiver Operating Curve (AUROC) characteristics with 95% confidence intervals for 5-year follow-up in the general population, age 40-64 years free from cancer at baseline are shown: From left to right, for all malignancies (ICD-10: C00-C97), Colorectal (C18-C21), Lung (C34), Melanoma (C43), Breast (C50) and Prostate (C61) with a 1-, 3- and 5-year look-back period for diagnoses, from top to bottom, for the Nordic Multimorbidity Index (NMI), the Charlson Comorbidity Index (CCI), the Elixhauser Comorbidity Index (ECI), numbers of distinct 3-character ICD-10 codes of main diagnoses, numbers of hospitalizations (visits), numbers of days hospitalized for in-patient care in the upper panel. Corresponding, with a 1-year look-back period for numbers of distinct 1-, 3-, 4-, 5- and 7-character ATC-codes on filled prescriptions are presented in the lower panel.

Models were based on (a) age-and-sex alone (The red line in each panel represents AUROC for age-and-sex), (b) index or measure alone (light blue symbols), and (c) age-and-sex *and* index, i.e. full model (dark blue symbols).

The 95%-Confidence intervals are, as seen very narrow, for details se the appendix tables 1-18.

Figure 3, upper panel

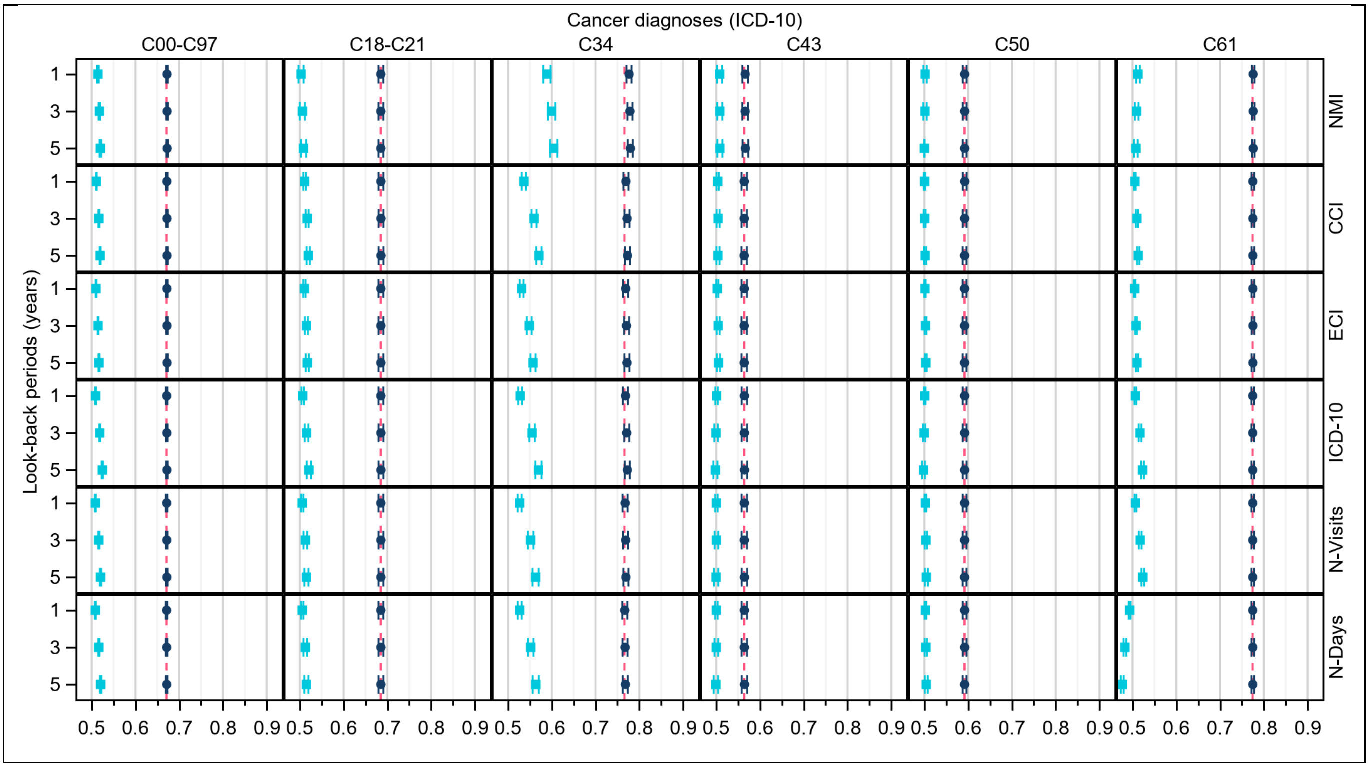

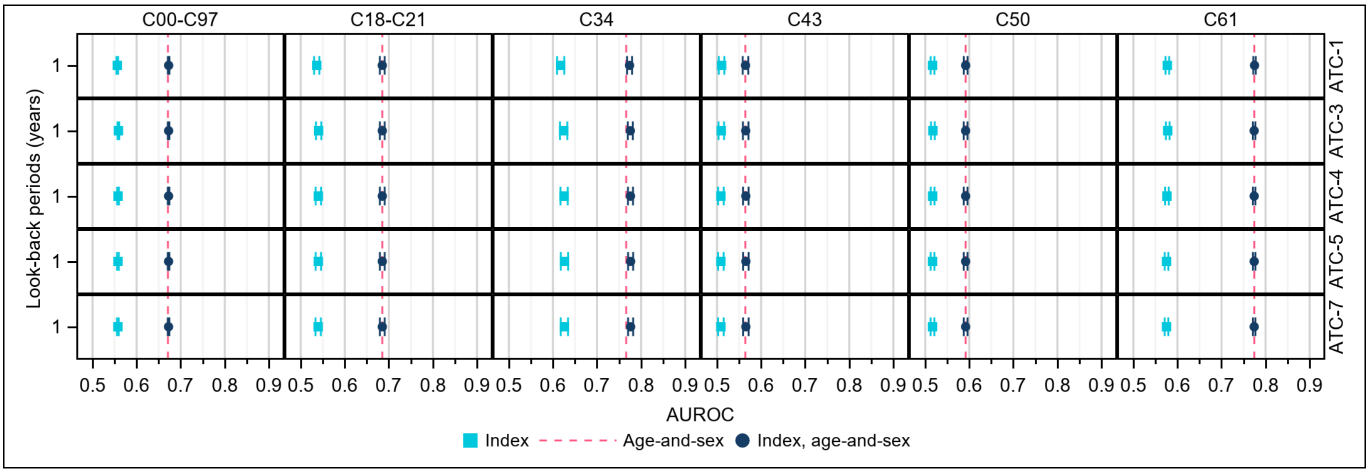

Figure 3, lower panel
